# Towards Implementation of Equitable and Effective Non-Communicable Disease Policies: Comment on "Barriers and Opportunities for WHO ‘Best Buys’ Non-Communicable Disease Policy Adoption and Implementation From a Political Economy Perspective: A Complexity Systematic Review"

**DOI:** 10.34172/ijhpm.9064

**Published:** 2025-10-14

**Authors:** Valerie A. Luyckx, Randall Lou-Meda

**Affiliations:** ^1^University Children’s Hospital, University of Zurich, Zurich, Switzerland.; ^2^Department of Public and Global Health, Epidemiology, Biostatistics and Prevention Institute, University of Zurich, Zurich, Switzerland.; ^3^Renal Division, Brigham and Women’s Hospital, Boston, MA, USA.; ^4^Harvard Medical School, Boston, MA, USA.; ^5^Department of Paediatrics and Child Health, University of Cape Town, Cape Town, South Africa.; ^6^Kidney Health Program, Ministry of Public Health and Social Assistance, Guatemala City, Guatemala.

**Keywords:** NCDs, Best Buys, Implementation Research, Priority Setting, Equity, Disease Burden

## Abstract

Non-communicable diseases (NCDs) have been the leading global causes of death and disease burden over the past two decades, but policies and actions to reduce these burdens have been insufficient. Many NCDs are preventable through the implementation of the World Health Organization (WHO) Best Buys – which initially focused on cardiovascular disease, cancer, respiratory disease, and diabetes. Implementing these interventions is complex, requiring transparent and appropriate policy development, policy implementation, and tracking of impact. Barriers to successful implementation are multiple and highly contextual, suchcountry fragility, loci of power, and external pressures. Implementation research is required to identify local barriers and develop strategies to optimize policy implementation to maximize success. Success relies on availability of robust data to permit priority setting, especially where resources are limited, and equitable allocation of healthcare resources to tackle the leading burdens of disease in local contexts. Policy-making must look beyond health to ensure a multisectoral approach to enhancing well-being and sustainability. Global solidarity is required to ensure no countries and no diseases are left behind.

 The COVID-19 pandemic woke the world up to the importance of non-communicable diseases (NCDs), not only as afflictions themselves but also as markers of social vulnerability and as barometers that mirror the resilience of health systems.^1^ In 2023, NCDs killed over 43 million people, accounting for 75% of all deaths, 73% of which occurred in low- and middle-income countries (LMICs).^2^ These realities highlight the complex interdependencies of social and political contexts and structural violence on health and well-being. However, despite these facts NCDs continue not to be proportionately prioritized on the global health agenda. The reasons for this oversight are complex, including lack of financing, donor agendas, pervasive global inequities, and the complexity inherent in preventing and managing NCDs.

 Financing for global health is a major driver of action. Given the focus on human immunodeficiency virus, tuberculosis, malaria, and maternal and child health set out by the Millennium Development Goals^3^ in 2000, financing and donor attention has centred on tracking and tackling the burdens of these conditions, while leaving many others behind. Also, with concerns around global health security, colonial powers traditionally focused support on “tropical diseases” in LMICs, and this prioritization has persisted to contain threats of spread of infectious diseases from the global south. Indeed, although NCDs comprise the bulk of the global disease burden in terms of deaths and disease-adjusted life years, over the past 25 years, only 2% of development aid to LMICs has been allocated towards NCDs.^4^ Telling also is the fact that more of these aid contributions towards NCDs come from private philanthropy rather than governments, reflecting the persistent relative lack of government prioritization of NCDs.^4^

 The disproportionate global allocation of funding towards infectious diseases also has a historical basis grounded in the concepts of “international health” or “global health.” Actors from the global north have focused on solving health problems—which they interpreted as priorities—afflicting the poor in the global south, largely through vertical programmes.^5^ One can plausibly understand that infectious diseases may seem relatively low hanging fruit in terms of improving population health. They are typically acute, need urgent treatment, tend to be cheap and simple to treat, are mostly curable, are contagious, afflict disadvantaged populations, and are also preventable. Crucial however, is also the fact that the impact of investment into targeted disease programmes is relatively straightforward to measure within a short time, and therefore from the donor perspective the “value for money” can be rapidly demonstrated. If one looks at these features critically, however, each one, with the exception of the length of time needed to assess programme impact given the chronicity of NCDs, could arguably apply equally to NCDs. NCDs can present acutely, require urgent treatment, may be curable, many treatments—especially if started early—are cheap and effective, and many are socially “contagious,” based on the ubiquitous commercial determinants of health and pervasive societal inequities. NCDs are also highly preventable, largely through improvement in poverty, nutrition, education and mitigation of social and structural determinants of health. The facts that addressing NCDs may require more effort than infectious diseases to tackle holistically, and that investments may not provide rapid impact results as incentives for donors, cannot morally or ethically justify the prevailing inequitable and unequal global approach to communicable and NCDs.^6^

 People living with, or at risk of, NCDs have the same rights as those at risk of, or living with infections to public health and preventive measures to protect their health. COVID-19 highlighted this very starkly – the narrow focus on tackling the infection led to many more excess deaths from health systems disruption and lack of planning to meet the needs of those with NCDs.^1^ This relative “neglect” of NCDs needs to change – additional and equitably distributed resources and sustainable strategies are required.

 In 2011, at the first United Nations High Level Meeting on NCDs, recognizing that NCDs have been the leading global causes of death for decades, Member States agreed to a target reduction of premature mortality from NCDs of 25% by 2025.^7^ Subsequently the Sustainable Development Goals (SDGs), launched in 2015, focused on health and well-being in SDG3, which included targeting premature deaths from NCDs. As acknowledged during the third United Nations High Level Meeting on NCDs in 2018, and in the report of the World Health Organization’s (WHO’s) High-Level Commission on NCDs, progress in reaching the NCDs targets has been insufficient.^7^ In 2019, the target was extended to reduce NCD deaths by one third by 2030.^8^ We are now in 2025, and according to the 2024 SDG Report, marginal progress has been achieved.^9^

 Tackling the NCD burden successfully requires holistic, comprehensive, and long-term strategies which include disease prevention and equitable access to early diagnosis and quality care under universal health coverage. In the 2024 SGD report, António Guterres is quoted as saying: “We have a rescue plan before us, in the [SDG Summit] political declaration. Now is the time to lift the declaration’s words off the page, and invest in development at scale like never before.”^9^

 With respect to the “rescue plan” for NCDs, four “priority” NCDs were identified in 2000—cardiovascular disease, cancer, respiratory disease and diabetes—with the justification that these contributed to the majority of premature NCD deaths.^10^ This utilitarian approach focused also on tackling four main risk factors for these four conditions (4 x 4 approach). To this end, the WHO’s Best Buys were identified as a set of cost-effective interventions, designed to address these four risk factors – tobacco consumption, harmful alcohol use, unhealthy diets and low physical exercise.^8^ As happens when a list of priorities is identified, the global efforts to tackle NCDs since 2008 have focused on these four priority conditions (with the more recent addition of mental health), which has resulted in the overlooking of many others (Figure). This inequitable utilitarian approach within NCDs themselves may make some sense at a policy-making level – to focus on what are considered to be the leading causes of death and morbidity – but comes at the cost of overlooking other important conditions. This prioritization approach relies heavily on data, therefore disease burdens that are unmeasured remain unseen. This approach also ignores the reality that many people with NCDs are living with multiple NCDs, and therefore focusing only on some of an individual’s clinical problems will not solve the whole.

**Figure:**
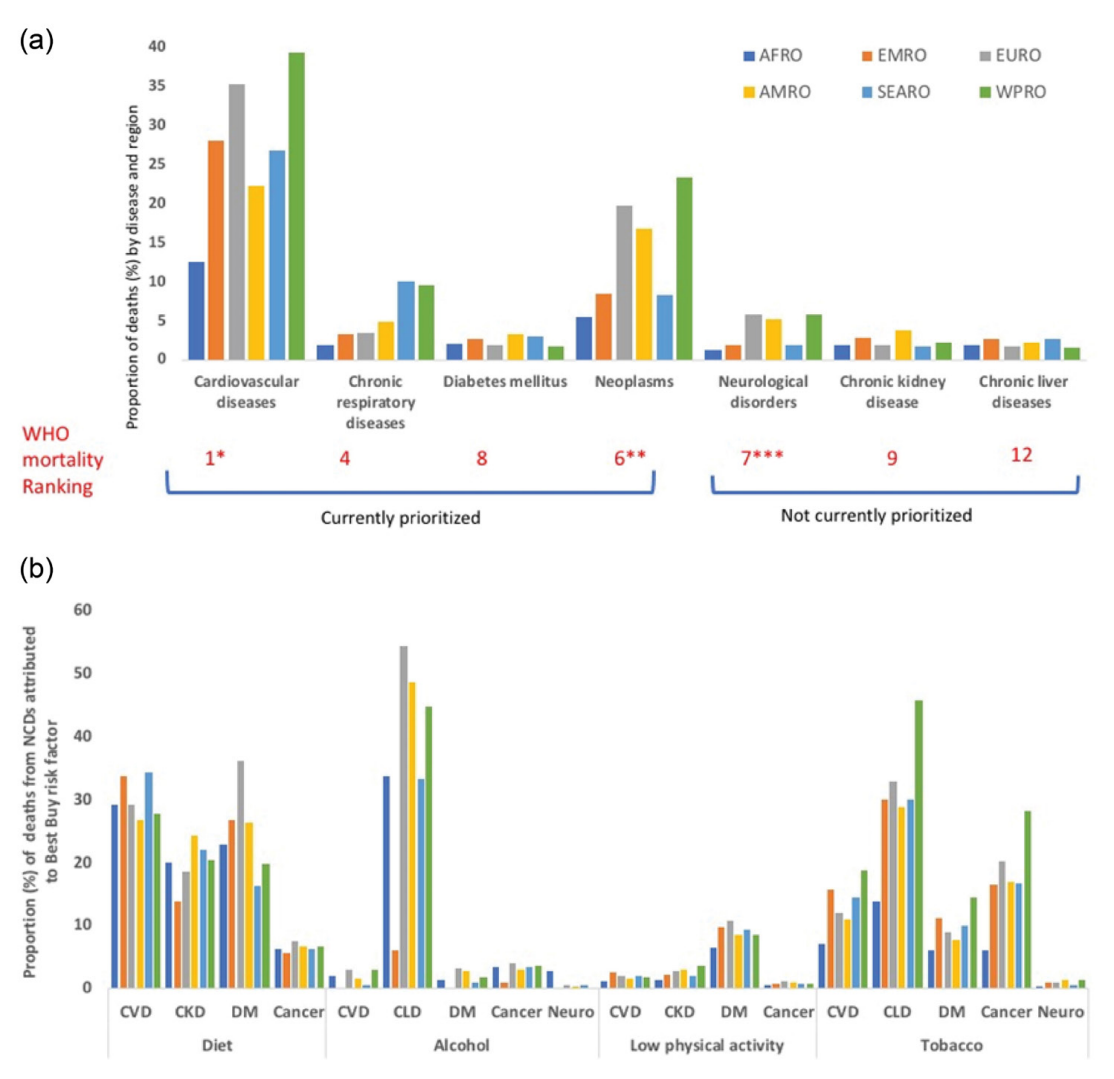
Proportion of Deaths (%) Attributed to Leading Non-communicable Diseases and the Best Buy Risk Factors, by WHO Region. (a) Proportion of deaths attributed to the four priority NCDs (cardiovascular diseases, chronic respiratory diseases, neoplasms, and diabetes). It is evident that other non-prioritized NCDs also contribute to a significant number of deaths as indicated in the figure by their 2021 rankings by WHO. (b) Proportion of deaths from leading NCDs attributed to the risk factors targeted by the WHO Best Buys. It is evident that the impact of these risk factors may vary by region. Local data is required to support appropriate local policy-making, to focus on risk factors for priority NCDs in local contexts. Abbreviations: NCDs, non-communicable diseases; WHO, World Health Organization; AFRO, WHO Regional Office for Africa; EMRO, WHO Regional Office for the Eastern Mediterranean; AMRO, WHO Regional Office for the Americas; WPRO, WHO Regional Office for the Western Pacific; EURO, WHO Regional Office for Europe; SEARO, WHO Regional Office for South-East Asia; CVD, cardiovascular disease; CKD, chronic kidney disease; DM, diabetes mellitus; CLD, chronic liver diseases; Neuro, neurological diseases. * Cardiovascular disease ranked 1^st^, Stroke ranked 3^rd^; ** WHO ranking for trachea, bronchus, and lung cancer; *** Alzheimers and other dementias. Data derived from: https://vizhub.healthdata.org/gbd-results/ and https://www.who.int/data/gho/data/themes/mortality-and-global-health-estimates.

 While the Best Buys which tackle risk factors are less utilitarian, and do indeed have the potential to prevent more than the 4 prioritized NCDs, it is clear that their implementation is far from optimal,^8^ given the inherent complexity of such interventions and multiple superimposed contextual factors in diverse settings. We need to move from theory to action.

 Barriers and opportunities which are relevant to lifting the words “off the page” and translating the “rescue plan” into action are the focus of the study by Loffreda et al.^11^ The authors performed a complex systematic review of 157 articles to identify political economy factors which influence adoption and implementation of NCD policies which relate to the WHO Best Buys. Three core variables, policy development and evolution, policy implementation, and impact tracking were identified as factors which support progress on NCDs. They identified several barriers to effective NCD policy implementation, which include lack of context-specific data, the need for enhanced multisectoral collaboration, and the need to limit the commercial determinants of health. They conclude that policy development is strongly impacted by contextual factors (eg, country fragility), world trade agreements, competing local priorities, available resources, social and cultural acceptability, the influence of external actors (eg, pressure from industry), the loci of power, and the opening of windows of opportunity. In addition, the implementation of laws to tackle NCDs (eg, food labelling, taxing unhealthy products), the financing, capacity and resilience of the health system, as well as their acceptability to the community, determine the success of policy implementation. Critical to the oversight and optimization of the process is the ongoing monitoring, evaluation and adaptation of the NCD policy implementation, to ensure that the desired goals are being achieved. As depicted in a causal loop diagram, Best Buy policy development and implementation is a highly complex system, the outcome of which will depend on the interplay between highly contextual enablers and barriers. The authors are to be congratulated on the depth and breadth of this analysis, which clearly identifies the needs for a considered, comprehensive and systematic approach to NCD policy implementation.

 Loffreda et al, rightly highlight the need for implementation research to identify contextual barriers and enable progress towards the implementation of the Best Buys, and to reduce the global burden of NCDs. As such, implementation research aims to translate what we know (evidence) into what and how we do (policy and practice) – to take known effective interventions “off the page,” and to optimize their implementation and uptake in new real-world settings. Implementation research must, however, be responsive to the needs of the communities where the research is to take place. Inherently, this process involves priority setting, which requires data, transparency and accountability on the part of the policy-makers, and trust and solidarity on the part of communities.

 In many LMICs today, the process of priority setting is severely hindered by a lack of robust data, and agendas are driven by funders and external priorities. As an example, with regards to NCDs, since 2008, global aggregate data has driven the 4 x 4 approach, which has been successful at the global level in reducing the burdens of cardiovascular diseases, chronic respiratory diseases and cancers, given the targeted programmes and tracking of disease burdens.^12^ This approach has, however, overlooked many other NCDs, the burdens of some of which, like kidney diseases and Alzheimer’s disease, are continuing to grow, and today fall within the top 5 causes of death in some regions (Figure).^12^ Data on disease burden and local inequities is required not only on a national level but also on a subnational level to facilitate tailoring of implementation of Best Buy policies to the needs of the local populations and tracking of effectiveness of policy implementation on the ground. Transparent data reporting also allows civil society to hold policy-makers accountable, reduce the potential for corruption, and support the process of policy optimization. Importantly also, cost-effectiveness data of many NCD interventions is lacking in many LMICs. Therefore, some highly effective interventions may have been overlooked in the list of Best Buys because of this.^13^ A practical way forward would be to support the implementation of regional health technology assessment centers in LMICs to allow the evaluation of context, evidence-based and efficient NCD policy development and implementation.

 From the individual perspective, NCDs themselves exacerbate disadvantage and vulnerability through chronic illness and worsening of poverty, loss of income, and catastrophic health expenditure, especially where care is not covered under universal health coverage.^14^ NCDs are, therefore, inherently complex and require a comprehensive and long-term view towards prevention and optimal management at the societal, public health, health systems and individual levels. The inextricable interdependencies of social and political context, power of external agendas, health systems capacity and resilience, population health literacy, and the need for accountability of policy-makers call for the transparent, locally tailored implementation of policies to reduce the global NCD burden, and the use of a human-rights based approach to ensure equity remains front and center in decision-making. A narrow focus on health will likely, however, not be enough. A Health in all Policies approach is required with a focus on justice, equity, and responsiveness across all sectors to mitigate the social and structural risk factors and barriers to care for NCDs. Now more than ever, with the unfavorable economic climate, there is an urgent need for global solidarity, to support countries everywhere to implement the Best Buys effectively and appropriately within their local contexts, not only to prevent NCDs and their complications, but to reduce overall health expenditure and improve economic productivity and well-being.^15^

## Ethical issues

 Not applicable.

## Conflicts of interest

 Authors declare that they have no conflicts of interest.
